# Detoxifying deoxynivalenol (DON)-contaminated feedstuff: consequences of sodium sulphite (SoS) treatment on performance and blood parameters in fattening pigs

**DOI:** 10.1007/s12550-019-00385-5

**Published:** 2020-01-20

**Authors:** L. Bahrenthien, J. Kluess, A. Berk, S. Kersten, J. Saltzmann, L. Hüther, D. Schatzmayr, H. E. Schwartz-Zimmermann, A. Zeyner, S. Dänicke

**Affiliations:** 1 Institute of Animal Nutrition, Friedrich-Loeffler-Institut (FLI), Federal Research Institute for Animal Health, Bundesallee 37, 38116 Braunschweig, Germany; 2 BIOMIN Holding GmbH, BIOMIN Research Center, Technopark 1, 3430 Tulln, Austria; 3 Christian Doppler Laboratory for Mycotoxin Metabolism, Institute of Bioanalytics and Agro-Metabolomics, Department of Agrobiotechnology (IFA-Tulln), University of Natural Resources and Life Sciences, Konrad Lorenz Straße 20, 3430 Tulln, Vienna Austria; 4 Institute of Agricultural and Nutritional Sciences, Martin-Luther-University Halle-Wittenberg, Theodor-Lieser-Straße 11, 06120 Halle, Germany

**Keywords:** Detoxification, Deoxynivalenol, Sodium sulphite, Fattening pigs, Performance

## Abstract

A 10-week feeding experiment was carried out examining the effects of deoxynivalenol (DON)-contaminated maize treated with different sodium sulphite (SoS) concentrations on performance, health and DON-plasma concentrations in fattening pigs. Two maize batches were used: background-contaminated (CON, 0.73 mg/kg maize) and *Fusarium*-toxin contaminated (DON, 44.45 mg/kg maize) maize. Both were wet preserved at 20% moisture content, with one of three (0.0, 2.5, 5.0 g/kg maize) sodium sulphite concentrations and propionic acid (15%). Each maize batch was then mixed into a barley-wheat-based diet at a proportion of 10%, resulting in the following 6 feeding groups: CON− (CON + 0.0 g SoS/kg maize), CON2.5 (CON + 2.5 g SoS/kg maize), CON5.0 (CON + 5.0 g SoS/kg maize), DON- (DON + 0.0 g SoS/kg maize), DON2.5 (DON + 2.5 g SoS/kg maize) and DON5.0 (DON + 5.0 g SoS/kg maize). Dietary DON concentration was reduced by ~ 36% in group DON2.5 and ~ 63% in group DON5.0. There was no impact on ZEN concentration in the diets due to SoS treatment. Pigs receiving diet DON- showed markedly lower feed intake (FI) compared to those fed the control diets. With SoS-treatment of maize, FI of pigs fed the DON diet (DON5.0: 3.35 kg/d) were comparable to that control (CON−: 3.30 kg/day), and these effects were also reflected in live weight gain. There were some effects of SoS, DON or their interaction on serum urea, cholesterol and albumin, but always within the physiological range and thus likely negligible. SoS wet preservation of *Fusarium*-toxin contaminated maize successfully detoxified DON to its innocuous sulfonates, thus restoring impaired performance in fatteners.

## Introduction

Deoxynivalenol (DON) and zearalenone (ZEN) are *Fusarium*-derived mycotoxins and are frequently co-occurring in cereals, especially in maize, barley and wheat. These cereals are often used as major components of diets for fattening pigs, and mycotoxin exposure can therefore pose a risk to pig production. Problems in animal production mainly occur due to a decreased feed intake (FI) and live weight gain (LWG) caused by the very sensitive response of pigs to DON exposure (Dänicke et al. 2004; Pestka 2007; Pierron et al. 2016; Reddy et al. 2018). Besides their negative impact on the performance of pigs, mycotoxins can have further adverse consequences. DON is known to inhibit protein synthesis, while ZEN possesses estrogenic properties of an endocrine disruptor (Döll and Dänicke 2011).

As the occurrence of DON in cereal grains cannot be averted completely, the need for management strategies of contaminated cereal batches arises. Besides disposal or blending of contaminated feed stuff with uncontaminated feed materials, there is the opportunity of decontaminating the feed. Various studies investigated decontamination methods applicable before feeding (physical and chemical treatment) or during feeding (in the digestive tract, e.g. microbial degradation) (Awad et al. 2010; He et al. 2010; Young 1986). It was revealed that chemical detoxification of DON-contaminated cereals with sulphur salts was successful (Dänicke et al. 2005; Paulick et al. 2015a; Tran et al. 2018a; Young et al. 1987). Young et al. (1987) described a considerable DON reduction after autoclaving contaminated corn in the presence of sodium sulphite. Dänicke et al. (2005) presented a 96% reduction of DON concentration by hydrothermal treatment of contaminated wheat with sodium metabisulphite (SBS). Schwartz et al. (2013) demonstrated that detoxification of DON is accompanied by formation of three structurally different, less toxic sulfonated DON derivatives, called DON-sulfonates (DONS1, DONS2 and DONS3). Paulick et al. (2015a) investigated effects of sodium sulphite (SoS) treatment on DON reduction and impact of increasing DONS concentrations in maize. A study with piglets showed that feeding a diet with SoS-treated DON-contaminated maize yielded performances comparable to the uncontaminated control diet (Paulick et al. 2018). However, efficacy of long-term use and possible side effects of the method are not yet clarified. In this context a study of Til et al. (1972) needs to be considered. The authors examined the toxicity of sulphite in a feeding experiment with an SBS treated diet for pigs. Usually, sulphite is applied as a preserving agent in food and drinks. Til et al. (1972) could not detect effects on health and mortality of pigs, but thiamine levels in urine and liver of pigs fed the sulphite diet were decreased in their investigation. Previous studies have only investigated one SoS concentration for detoxification tests; therefore, it would be interesting to evaluate the effects of different SoS dosages. To investigate the above-mentioned aspects a long-term *in vivo* study was designed to examine the effects of a constant proportion of wet-preserved *Fusarium*-toxin contaminated maize treated with graded SoS levels in the diet for fattening pigs.

## Materials and methods

The experiment was conducted at the Institute of Animal Nutrition, Braunschweig, Germany in agreement with the European Community regulations concerning the protection of experimental animals and the guidelines of the German Animal Welfare Act and was approved by the Lower Saxony State Office for Consumer Protection and Food Safety (file number: 33.19–42502–04-16/2325).

### Experimental diets

Two different maize batches, background-contaminated (CON) and *Fusarium*-toxin contaminated (DON) maize, generated by artificial inoculation with *Fusarium graminearum* spores as described in Paulick et al. (2015a) and Paulick et al. (2018) were used for the present feeding experiment. Both maize batches were wet-preserved for 63–70 d with one of three SoS concentrations (0.0, 2.5, or 5 g SoS/kg maize kernels), treated with 15 g propionic acid/kg maize at 20% moisture content. Both chemicals were purchased from Carl Roth GmbH & Co KG (Karlsruhe, Germany): Na_2_S_2_O_5_ (CAS-no. 7757-83-7, **≥** 98%, p.a., ACS, water free) and propionic acid (CAS-no. 79–09-4, 99.5% for synthesis). In order to maintain equal preservation periods (63-70d) we incubated three times per fattening phase (starter, grower, finisher phase) and maize batch in a time-dependent manner. After each round of preservation, maize batches were directly included at a proportion of 10% in a barley-wheat based diet, resulting in six dietary groups: **CON−** (CON + 0.0 g SoS/kg maize), **CON2.5** (CON + 2.5 g SoS/kg maize), **CON5.0** (CON + 5.0 g SoS/kg maize), **DON−** (DON + 0.0 g SoS/kg maize), **DON2.5** (DON + 2.5 g SoS/kg maize) and **DON5.0** (DON + 5.0 g SoS/kg maize). Each finished feed batch was used directly after production in the according feeding phase. Diets were formulated to meet or exceed animal requirements as recommended (GfE 2008) and composition of each fattening phase is provided in S1–3.

### Animal experiment

**Fig. 1:**
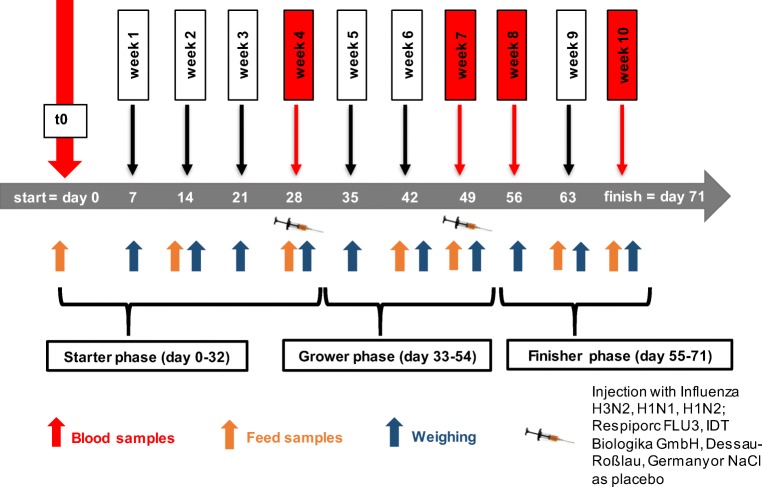
Experimental setup

The present study was carried out with a total of 96 barrows, originating from a breeding facility (Bundeshybridzuchtprogramm, Agrar-Genossenschaft, Lückstedt/Germany) with a high sanitary status (antibody-negative for PRRS-virus, *Mycoplasma hyopneumoniae*, *Actinobacillus pleuropneumoniae*, *Salmonella spp*.). Pigs were housed individually in floor pens with free access to water (nipple drinker) and feed. They were allowed to adjust to the new environment for 5 days after arrival and fed the control diet (CON−, starter phase) during this time. Figure 1 displays the experimental setup over a period of 10 weeks. Pigs were weighed (32.5 ± 3.4 kg) at day 0 (t0) and equally distributed over the six dietary treatments (*n* = 16/treatment) based on their individual body weight (BW). Dietary transition between fattening phases followed live weight development and duration of each phase is given in Fig. 1. Body weight and feed refusals were recorded individually once a week and LWG, FI and feed:gain ratio (F:G) were calculated. To investigate the response of the adaptive immune system, each dietary treatment group was bisected, and pigs were either vaccinated intramuscularly with a commercial influenza vaccine (Respiporc FLU3, IDT Biologika GmbH, Dessau-Roßlau, Germany; strains: H3N2, H1N1, H1N2) or injected with a placebo (0.9% NaCl, sterile, equivolumar). Injections were performed on days 28 and 49 (booster vaccination) directly after blood sampling. Full vaccination was thus accomplished during the finisher phase. Blood samples were collected via puncturing the neck vessels at days 0 (t0), 28, 49, 56 and 71 (end of trial). Data investigating the development of influenza titres are not part of this publication.

### Analyses

#### Nutrient and mycotoxin analyses in diets

Every 14 days, feed samples were taken for the analyses of nutrients, pooled for each fattening phase and diet and then ground to pass through a 1-mm sieve prior to analysing the chemical composition. The following methods of the Association of German Agricultural Analytic and Research Institutes (VDLUFA-Methodenbuch III 2004) were used: dry matter (3.1), crude ash (8.1), crude protein (Dumas-method, 4.1.2), crude fat (5.1.1) and crude fibre (6.1.1). Results of pooled feed samples are presented as mean value per fattening phase.

For mycotoxin-analyses, feed samples were collected every 14 days. Concentrations of DON, DONS and ZEN in diets were determined by ultra-high performance liquid chromatography (RP-UHPLC) coupled with tandem mass spectrometry (MS/MS) using a combination of two previously published methods. The used UHPLC system, column and mobile phases are described in Schwartz-Zimmermann et al. (2017), the sample preparation method and the selected reaction monitoring (SRM) transitions for determination of the three DONS are published in Schwartz-Zimmermann et al. (2014). The UHPLC gradient was as follows: 0.0–0.5 min: 5% B, 0.5–7.0 min: linear increase to 15% B, 7.0–17.0 min: linear increase to 100% B, 17.0–18.0 min: 100% B, 18.1–21.0 min: 5% B. The flow rate was 0.25 mL/min, and the injection volume was 3 μL. The SRM transitions for DON were declustering potential (DP) = −70 V, quantifier = *m/z* 355.1− > *m/z* 59.0 (collision energy, CE − 38 eV), qualifier = *m/z* 355.1− > *m/z* 265.0 (CE − 18 eV). The SRM transitions for ZEN were: DP -120 V, quantifier = *m/z* 317.1- > *m/z* 131.1 (CE −42 eV), qualifier = *m/z* 317.1− > *m/z* 175.1 (CE − 34 eV). Limit of detection (LOD) and limit of quantification (LOQ) in feed are provided in Table 1.

**Table 1:** The limit of detection (LOD) and quantification (LOQ) of deoxynivalenol (DON), zearalenone (ZEN) and their metabolites

			DON-Sulfonates						
		DON	S1^*^	S2^*^	S3^*^	DOM-1^°^	ZEN	α-ZEL^#^	β-ZEL^†^
**Feed**	LOD (mg/kg)	0.074	0.019	0.011	0.056	–	0.011	–	–
	LOQ (mg/kg)	0.247	0.062	0.037	0.185	–	0.037	–	–
	Recovery (%)	81	106	91	98		75		
**Plasma**	LOD (ng/mL)	0.22	0.35	0.25	0.86	0.16	0.02	0.13	0.18
	LOQ (ng/mL)	0.72	1.16	0.83	2.88	0.55	0.08	0.42	0.59
	Recovery (%)	112	116	78	70	70	112	94	114

*DON-sulfonates, °de-epoxy-DON, ^#^α-zearalenol, ^†^ß-zearalenol

#### Mycotoxin analyses in blood plasma

DON, ZEN and their respective metabolites were determined in heparinized plasma (S-Monovette®, Lithium-Heparin, Sarstedt AG & Co., Sarstedt, Germany) collected at day 71 (end of trial). Samples were measured with HPLC-MS/MS, using an Agilent 1200 series HPLC system (Agilent Technologies, Böblingen, Germany) coupled to a 4000 QTrap mass spectrometer (SCIEX, Foster City, CA, USA) described in Paulick et al. (2015b). Analysis of DON, de-epoxy-DON (DOM-1), ZEN and its metabolites was performed according to Brezina et al. (2014) and of DONS1, 2 and 3 as described by Paulick et al. (2018). Limit of detection (LOD) and limit of quantification (LOQ) in plasma are provided in Table 1.

#### Clinical biochemistry

Blood samples of day 0, 28 and 71 (end of trial) were collected in serum tubes (Serum Z, Sarstedt AG&Co, Sarstedt, Germany), allowed clotting for 60 min at room temperature and then centrifuged at 2123 g for 15 min (15 °C), aliquoted and stored at − 80 °C until analyses. Serum was analysed for total protein, albumin, urea, aspartate-aminotransferase (AST), alanine-aminotransferase (ALT), γ-glutamyltransferase (γ-GT), total bilirubin, alkaline phosphatase (ALP), triglycerides, cholesterol and glucose using photometric methods with an automatic analyser (Eurolyser, CCA180, Eurolyser Diagnostika GmbH, Salzburg, Austria).

#### Thiamine

Thiamine concentrations were analysed in heparinized plasma (S-Monovette®, Lithium-Heparin, Sarstedt AG & Co., Sarstedt, Germany; day 0, 28 and 71) at LABOKLIN (LABOKLIN GmbH&Co.KG, 97688 Bad Kissingen) by using HPLC with a thiamine test kit. The limit of detection (LOD) for thiamine was 0.5 μg/L in plasma.

#### Sulphate

Sulphate concentration in plasma of pigs at the end of the experiment (day 71) was determined by turbidimetric method according to United States Enviromental Protection Agency (EPA) and American Public Health Association (APHA) with additional automation by use of a flow injection analyser (MLE GmbH, Dresden, Germany). In brief, plasma samples were diluted with doubly distilled water, and after addition of a barium chloride solution, plasma sulphate ions were converted to a barium sulphate suspension. The resulting turbidity was determined by a filter photometer at 460 nm and compared to a curve prepared from standard sulphate solutions in the range of 5–100 μg/mL. Turbidity due to the sample matrix can cause positive interferences which were captured by use of blanks (APHA 1992; EPA 1986).

### Statistical analysis

Parameters were statistically analysed with the procedure MIXED (SAS 9.4) using a restricted maximum likelihood model (REML).

For starter and grower phase, performance parameters were statistically analysed using a two-factorial design with maize (DON vs. CON) and SoS treatment (0, 2.5 and 5.0 g SoS/kg maize) as fixed factors and their interaction (DON*SoS). In the finisher phase, however, a three-factorial design was used with vaccination (VACC; influenza vs. NaCl) as additional fixed factor and the following interactions: DON*SoS, DON*VACC, SoS*VACC, DON*SoS*VACC. The adjusted Tukey-Kramer was applied as *post hoc* test in case of statistical significances of main factors (*p* < 0.05). Results are presented as least square means (LSMeans) with pooled standard error of means. The three-factorial design was also employed for parameters of clinical biochemistry and thiamine concentration, but with t0 as covariable and time as a repeated measure. Maize (DON vs. CON), SoS treatment (0, 2.5 and 5.0 g SoS/kg maize) and vaccination (Influenza vs. NaCl) were used as fixed factors whereby also their interactions (DON*SoS, DON*Time, SoS*Time, DON*SoS*Time) were included. Because vaccination of pigs took place only in week 4 and 7, factor VACC was not included in the interactions. Data for DON, ZEN and their metabolites in plasma were first tested for outliers. Because they did not follow a Gaussian distribution, they were evaluated with a Kruskal–Wallis test (Statistica 64, TIBCO Software Inc., USA). Values below LOD were treated as zero, while values between LOD and LOQ are presented at their determined value.

## Results

### Experimental diets

All diets over all three fattening phases were isoenergetic and isonitrogenous. Additionally, they complied with the requirements for each fattening phase (S1–3) as recommended by the GfE (2008).

In groups CON−, CON2.5 and CON5.0, concentration of DON was < 0.05 mg/kg feed and of ZEN < 0.01 mg/kg feed in diets for all three fattening phases. Concentrations of DONS1 and DONS3 were lower than LOD in all CON groups (with and without SoS), but DONS2 occurred in traces (0.04 mg/kg) in the CON groups treated with SoS in starter and grower phase. The mean DON concentration in group DON− was 4.4 mg/kg in all fattening phases, but decreased with increasing SoS-concentration (DON2.5, DON5.0). This was accompanied by an increase in concentrations of DON-sulfonates (Table 2).

**Table 2:** Analysed mycotoxin concentration in experimental diets and calculated daily mycotoxin dose in all fattening phases

	Mycotoxin concentration in diets (mg/kg diet)						Mycotoxin dose (μg/kg BW)		
	CON-	CON2.5	CON5.0	DON-	DON2.5	DON5.0	DON-	DON2.5	DON5.0
Starter phase									
DON	< 0.05	< 0.05	< 0.05	3.88	3.23	1.44	188.67	171.44	76.31
DONS1	< 0.02	< 0.02	< 0.02	< 0.02	0.05	0.07	0.00	2.43	3.67
DONS2	< 0.02	< 0.02	0.04	< 0.01	1.12	1.59	0.00	59.64	84.31
DONS3	< 0.02	< 0.02	< 0.02	< 0.04	1.26	1.32	0.00	67.12	70.05
ZEN	< 0.01	< 0.01	< 0.01	0.27	0.41	0.46	13.24	21.81	24.29
Grower phase									
DON	< 0.05	< 0.05	< 0.05	4.69	2.93	1.74	210.78	134.96	81.46
DONS1	< 0.02	< 0.02	< 0.02	< 0.02	0.06	0.05	0.00	2.63	2.58
DONS2	< 0.02	0.04	0.04	< 0.01	1.42	1.44	0.00	65.28	67.49
DONS3	< 0.02	< 0.02	< 0.02	< 0.04	1.12	1.18	0.00	51.73	55.24
ZEN	< 0.01	< 0.01	< 0.01	0.33	0.33	0.30	14.77	15.39	14.12
Finisher phase									
DON	< 0.05	< 0.05	< 0.05	4.44	2.75	1.45	134.30	87.25	46.58
DONS1	< 0.02	< 0.02	< 0.02	< 0.02	0.05	0.06	0.00	1.62	1.95
DONS2	< 0.02	< 0.02	< 0.02	< 0.01	1.49	1.42	0.00	47.27	45.61
DONS3	< 0.02	< 0.02	< 0.02	< 0.04	0.52	0.59	0.00	16.38	18.88
ZEN	< 0.01	<0.01	< 0.01	0.25	0.36	0.28	7.56	11.34	9.03

**Fig. 2:**
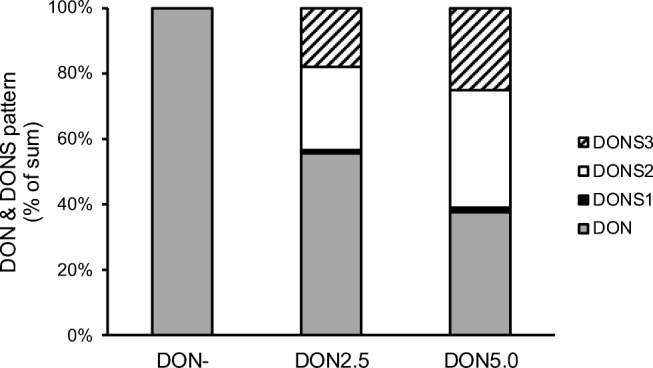
Relative distribution of deoxynivalenol and its sulfonates DONS1, DONS2 and DONS3 in compound diets (% sum of DON and DON-sulfonates), containing *Fusarium*-toxin contaminated maize treated with three different levels of SoS (0.0, 2.5, 5.0 g/kg maize). Data of each fattening phase (starter, grower, finisher) were averaged over the entire fattening period as there were no differences in distribution pattern between phases

Figure 2 presents the proportions of DON and its sulfonates in per cent of their sum in DON-contaminated groups. With higher amounts of SoS, the proportions of DONS were increasing, whereas DON concentration decreased.

ZEN concentration in all fattening phases was not affected by SoS-treatment. Mean concentrations of ZEN were < 0.01 mg/kg in CON diets and 0.36 mg/kg in DON diets.

### Animal performance and daily mycotoxin dose

Four pigs had to be excluded from the experiment because of rectal prolapse (*n* = 3; groups CON5.0, DON-, DON5.0) and a broken leg (*n* = 1; group CON5.0).

Data on animal performance and oral mycotoxin dose are detailed in Table 2 and Table 3, respectively. In starter and grower phase, pigs of group DON− showed markedly lower FI compared to their CON-fed counterparts. Pigs fed the diets containing SoS-treated, *Fusarium-*toxin contaminated maize (DON2.5, DON5.0) showed FI comparable with pigs of control groups, which in turn did not differ between each other. Thus, a significant interaction between DON*SoS was proven for FI in the starter and grower phase as well as for the overall trial period.

**Table 3:** Performance parameters of pigs fed diets over 71 days containing control (CON) or *Fusarium*-toxin contaminated maize (DON) treated with one of three sodium sulphite (SoS) concentrations: (CON-, 0.0 g SoS/kg CON maize; CON2.5, 2.5 g SoS/kg CON maize; CON5.0, 5.0 g SoS/kg CON maize, DON-, 0.0 g SoS/kg DON maize; DON2.5, 2.5 g SoS/kg DON maize; DON5.0, 5.0 g SoS/kg DON maize) in a 3-phase feeding regimen (LSMeans)

	Feed intake (kg/day), FI				Live weight gain (kg/day), LWG				Feed:Gain ratio, F:G			
	ST	GR	F	overall	ST	GR	F	overall	ST	GR	F	overall
Group	(*n* = 96)	(*n* = 94)	(*n* = 92)		(*n* = 96)	(*n* = 94)	(*n* = 92)		(*n* = 96)	(*n* = 94)	(*n* = 92)	
CON-	2.79	4.05	3.30	3.30	1.26	1.29	1.15	1.24	2.24	3.17	2.91	2.68
CON2.5	2.76	4.00	3.48	3.32	1.21	1.27	1.14	1.20	2.30	3.16	3.14	2.76
CON5.0	2.71	4.00	3.45	3.28	1.22	1.27	1.13	1.20	2.23	3.15	3.06	2.73
DON-	2.44	3.56	3.05	2.91	1.09	1.15	1.05	1.10	2.25	3.14	2.99	2.65
DON2.5	2.78	3.85	3.37	3.25	1.20	1.22	1.19	1.20	2.33	3.19	2.86	2.71
DON5.0	2.79	4.04	3.52	3.35	1.27	1.29	1.21	1.26	2.21	3.14	2.93	2.66
F-Test (*p*values)												
DON	0.115	0.006	0.140	0.017	0.114	0.029	0.624	0.203	0.856	0.967	0.145	0.277
SoS	0.031	0.051	0.001	0.004	0.078	0.164	0.248	0.104	0.262	0.922	0.879	0.362
VACC^#^	–	–	0.454	0.272	–	–	0.265	0.331	–	–	0.501	0.920
DON*SoS	0.002	0.010	0.163	0.002	0.001	0.079	0.052	0.002	0.924	0.939	0.157	0.961
DON*VACC	–	–	0.558	0.214	–	–	0.846	0.390	–	–	0.814	0.696
SoS*VACC	–	–	0.691	0.612	–	–	0.036	0.174	–	–	0.050	0.634
DON*SoS*VACC	–	–	0.696	0.441	–	–	0.747	0.563	–	–	0.878	0.985
PSEM*	0.06	0.09	0.08	0.06	0.03	0.03	0.04	0.03	0.06	0.08	0.09	0.05

ST = starter, GR = grower, F = finisher, *pooled standard error of means, ^**#**^vaccination: only parameters in finisher phase included VACC as a fixed factor

The daily mycotoxin dose (mg/kg BW) of pigs receiving the three DON-diets was calculated as an average over each fattening phase, taking FI and body weight into account (Table 2). The positive impact of increasing SoS-dosage in maize-treatment is clearly detectable in each fattening phase, whereby the most pronounced decrease in daily DON-dose was apparent in group DON5.0. Furthermore, with a decline in DON-uptake we saw a concomitant rise in DONS-doses, particularly DONS2 and DONS3. Daily ZEN-doses were overall not affected by SoS-treatment of maize.

LWG reflected the effects observed for FI. Here, the interaction between DON and SoS was significant in starter and finisher phase. LWG decreased in CON groups when maize was treated with SoS, whereby LWG increased in groups fed the DON-maize treated with SoS. Additionally, an interaction between SoS and VACC was detected for LWG in the finisher phase. Here, LWG marginally decreased in pigs injected with the placebo and fed with the SoS-treated maize. In contrast, LWG increased in pigs vaccinated with influenza in connection with SoS treatment. Because FI and LWG showed similarly directed effects, there were no changes for F:G.

### Mycotoxin concentrations in blood

The mean concentrations of DON, DOM-1, ZEN and its metabolites in plasma (Table 4) of the CON groups were between LOD and LOQ, whereby concentrations of DONS were lower than the corresponding LOD. Mycotoxin residue values were comparable in all CON-groups. DON concentrations in plasma of DON groups were numerically decreased with increasing SoS-concentrations. In contrast to DONS1 and DONS3, concentrations of DONS2 were increased in the DON groups treated with SoS compared to group DON−. DOM-1 concentrations were not significantly different between DON groups, but higher compared to CON groups. Concentrations of α-ZEL were significantly higher in DON groups, but no significant differences in concentrations of ß-ZEL were determined.

**Table 4:** Concentration (median, minimum-maximum) of deoxynivalenol (DON), zearalenone (ZEN) and their metabolites in plasma (ng/mL) of pigs fed diets for 71 days containing control (CON-) or *Fusarium*-toxin contaminated maize (DON-) treated with one of three sodium sulphite (SoS) concentrations: CON-, 0.0 g SoS/kg CON maize; CON2.5, 2.5 g SoS/kg CON maize; CON5.0, 5.0 g SoS/kg CON maize, DON-, 0.0 g SoS/kg DON maize; DON2.5, 2.5 g SoS/kg DON maize; DON5.0, 5.0 g SoS/kg DON maize) in a 3-phase feeding regimen (LSMeans).

	CON-	CON2.5	CON5.0	DON-	DON2.5	DON5.0
DON	0.53 (0–0.70)^ac^	0.34 (0–0.55)^a^	0.35 (0–4.34)^a^	21.30 (9.20–30.30)^b^	17.10 (12.60–19.90)^b^	12.50 (3.20–14.20)^bc^
DONS1	0 (0–0)	0 (0–0)	0 (0–0)	0 (0–0)	0 (0–0)	0 (0–0)
DONS2	0 (0–0)^a^	0 (0–0)^a^	0 (0–0)^a^	0 (0–0)^a^	0.82 (0.37–1.64)^b^	1.23 (0.62–1.93)^b^
DONS3	0 (0–0)	0 (0–0)	0 (0–0)	0 (0–0)	0 (0–0)	0 (0–1.71)
DOM1	0 (0–0)^a^	0 (0–0)^a^	0 (0–0.29)^a^	3.6 (2.73–6.08)^b^	2.71 (1.12–4.00)^b^	2.09 (1.55–3.94)^b^
ZEN	0.03 (0–0.06)^a^	0.03 (0–0.07)^a^	0.05 (0–0.14)^a^	0.57 (0.28–1.10)^b^	0.72 (0.41–1.01)^b^	0.65 (0.38–0.89)^b^
α-ZEL^#^	0.14 (0–0.40)^a^	0.23 (0–0.42)^a^	0.17 (0.0–3.13)^a^	1.77 (0.58–2.90)^b^	2.29 (1.36–4.02)^b^	2.26 (1.49–3.15)^b^
ß-ZEL^†^	0 (0–0)	0 (0–0)	0 (0–0)	0 (0–0.16)	0 (0–0.31)	0.19 (0–0.81)

^#^α-zearalenol; ^†^ß-zearalenol, ^abc^data with different superscripts in the same line are significantly different from each other (Kruskal-Wallis-test, *p* < 0.05)

### Clinical biochemistry

#### Protein metabolism

**Fig. 3:**
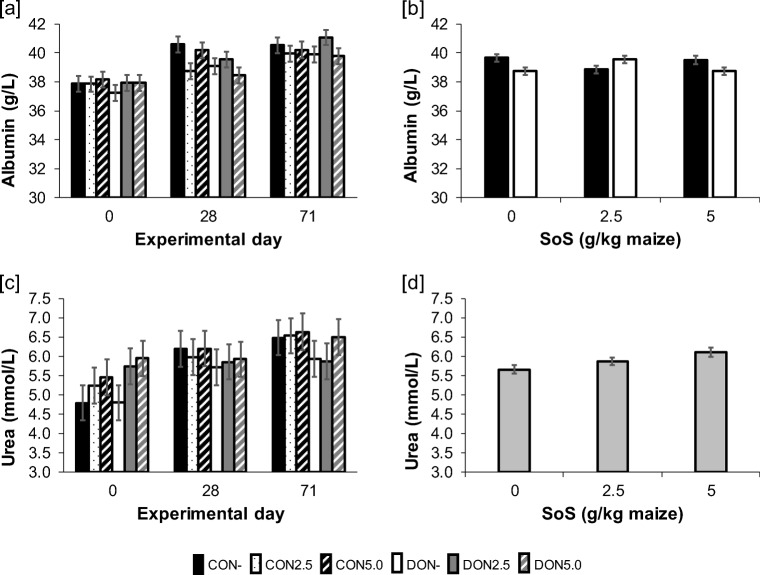
Time course (0, 28, 71 days of experiment) of serum [**a**] albumin and [**c**] urea in fattening pigs receiving diets containing control (CON-) or *Fusarium*-toxin contaminated maize (DON−) treated with one of three sodium sulphite (SoS) concentrations (0.0 SoS/kg maize, 2.5 g SoS/kg maize and 5.0 g SoS/kg maize). Data are presented as LSMeans ± SE and significant main effects comprise: [**b**] DON*SoS interaction (*p* = **0.005**) of albumin concentration (LSMeans ± SE); [d] SoS effect (*p* = **0.017**) of urea concentration (LSMeans ± SE)

All parameters (total protein, albumin, urea) were significantly affected by the main factor time (S4), steadily increasing from base levels at 0 day to 28 day and the final day 71. Albumin and urea also showed additional significant impacts that are displayed in Fig. 3. For albumin concentration, a significant interaction between DON and SoS (Fig. 3b) was determined, resulting from a decrease in CON2.5 and return to base levels in CON5.0 and a contrasting development in DON-groups. Urea was significantly increased with rising SoS dosage of maize (Fig. 3d), whereby the highest SoS concentration (5 g SoS/kg maize) differed significantly from the lowest (*post hoc* adj. Tukey-Kramer test: *p* > 0.05). Furthermore, urea concentration was significantly decreased in vaccinated pigs as compared to placebo-injected animals (VACC vs. placebo: 6.0 vs. 5.7 mmol/L).

#### Hepatic metabolism

All parameters (S5) of the hepatic metabolism (AST, ALT, AST/ALT ratio, γ-glutamyltransferase [γ-GT], total bilirubin, ALP) were significantly affected by the factor time, but in varying manners: AST decreased (41, 40, 30 nkat/L) and ALT increased (42, 58, 57 nkat/L) over time, resulting overall in a decreased ratio. Bilirubin (9.4, 7.9, 7.8 μmol/L) and ALP (3034, 2981, 2514 nkat/L) showed also a decline from 0 day to 71 days, whereas γ-GT showed a bell-shaped curve, peaking at 28 days and returning to initial values at 71 days (729, 932, 778 nkat/L). Additionally, ALP showed a significant increase in influenza-vaccinated pigs as compared to their placebo counterparts (VACC vs. placebo: 2762 vs. 2925 nkat/L).

#### Energy metabolism

**Fig. 4:**
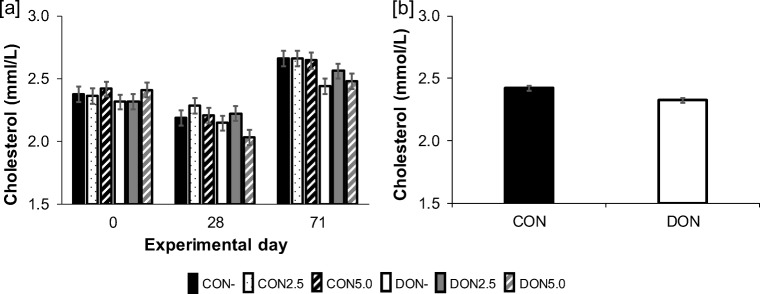
Time course (0, 28, 71 days of experiment) of serum [**a**] cholesterol in fattening pigs receiving diets containing control (CON) or *Fusarium*-toxin contaminated maize (DON−) treated with one of three sodium sulphite (SoS) concentrations (0.0 SoS/kg maize, 2.5 g SoS/kg maize and 5.0 g SoS/kg maize). Presence of DON lowered [**b**] cholesterol significantly (*p*_DON_ = 0.002; LSMeans ± SE)

Glucose, triglycerides and cholesterol as markers of energy metabolism (S6) were all significantly affected by time as main factor. Triglycerides declined over time (0 day: 0.63, 28 days: 0.41, 71 days: 0.45 mmol/L), whereas glucose levels peaked at 28 days (0 day: 5.28, 28 days: 5.48, 71 days: 5.10 mmol/L). In contrast, cholesterol (Fig. 4) showed its lowest value at 28 days (0d: 2.37, 28d: 2.18, 71d: 2.57 mmol/L). Besides this time effect, a significant DON effect was determined, whereby cholesterol concentration was lower in pigs fed diets with *Fusarium*-toxin contaminated maize (Fig. 4b) as compared to control.

#### Thiamine in plasma

**Fig. 5:**
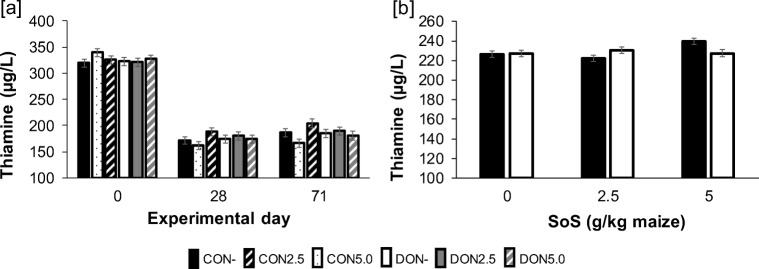
Time course [a] of thiamine concentration in plasma and [b] its significant DON*SoS interaction. Data represent LSMeans (± SE) and statistical effects were distributed as follows: *p*_DON_ = 0.711, *p*_SoS_ = 0.062, *p*_VACC_ = 0.627, *p*_Time_ = **< 0.001**, *p*_DON*SoS_ = **0.015**; *p*_DON*Time_ = 0.804 *p*_SoS*Time_ = 0.556, *p*_DON*SoS*Time_ = 0.061, *p*_CoV_ < 0.001

The time course of thiamine plasma concentration is depicted in Fig. 5a: thiamine was significantly decreased after 28 and 71 days as compared to initial values at 0 day. Moreover, a significant interaction between factors DON and SoS was also determined. This effect resulted from a significantly higher thiamine concentration in group CON5.0 as compared to CON2.5, whereas the other groups were not different from each other (Fig. 5b).

#### Plasma sulphate concentration

Treatments did not influence sulphate concentration in plasma. The overall mean plasma sulphate concentration amounted to 49.5 ± 5.5 μg/mL at day 71, the end of the trial.

## Discussion

Mycotoxin concentrations in diets and plasma of pigs were examined in order to investigate the impact of different sodium sulphite (SoS) concentrations on the decontamination of DON-contaminated maize during wet preservation. The results of the analysed mycotoxin concentrations in feed displayed a reduction of DON concentration in *Fusarium-*toxin contaminated diets treated with SoS. In the uncontaminated diets (CON) concentrations of DON, DONS1, DONS3 and ZEN were lower than LOD. Only for DONS2, traces were found in diets of the control groups. This can be explained by the small background concentration of DON in the control diets, which apparently resulted in a degradation of DON into DONS2 after 63 days of wet preservation. DON concentration was reduced by about 36% in diet DON2.5 and by about 64% in diet DON5.0. This outcome confirms the investigations of Paulick et al. (2015a) who observed an advantageous effect of SoS on DON reduction at concentrations greater than 2.5 g SoS/kg maize. In another study DON concentration in maize could be reduced by about 85% due to treatment with 5 g of SoS/kg maize (Paulick et al. 2018). In the current trial, the *Fusarium-*toxin contaminated diets treated with SoS exhibited increased concentrations of DONS2 and DONS3, whereby concentrations of DONS2 were 25.74% higher than concentrations of DONS3. Lower concentrations of DONS3 compared to DONS2 can be explained by the instability of DONS3 and the possible conversion of DONS3 back to DON and DONS2 due to a longer time of wet preservation (Paulick et al. 2018; Schwartz et al. 2013). DONS1 was not detectable in diet DON−, and only values in the range of the LOQ were detected in DON diets treated with SoS. These results confirm the investigations of Paulick et al. (2018) where DONS1 was not detectable in the SoS-treated DON diet. In the present study, the concentrations of ZEN were higher in *Fusarium-*toxin contaminated diets compared to the control diets. There were no remarkable differences between ZEN concentrations in DON diets in the context of SoS treatment. Considering the chemical structure of DON and the chemistry of the formation of sulfonates, similar effects were not to be expected for ZEN, which is completely different in structure and further chemical features. The inefficacy of the inactivation procedure with regard to ZEN was similarly reported for the related compound SBS (Dänicke et al. 2010). Conversion of DON into its sulfonates in the diets caused by SoS treatment was also reflected in the blood analyses, although here the magnitude of DON reduction due to SoS-treatment (compared to group DON−) was not as high as in the diets. Compared to the reduction in feed, DON reduction in plasma for group DON2.5 and DON5.0 were only 19.7% and 41.3%, respectively.

A key factor in the formation of DONS is the pH value of the corresponding medium. DONS3 is mainly formed in a slightly acidic to neutral environment, while DONS2 has its optimal formation from neutral to alkaline conditions (Schwartz et al. 2013). In our experimental diets, containing *Fusarium*-contaminated maize treated with increasing concentration of SoS, DONS2 (mean value: 1.4 ± 0.2 mg/kg feed) and DONS3 (mean value: 1.0 ± 0.3 mg/kg feed) concentrations were similar. Based on the before-mentioned pH optima, we can assume that DONS3 was mainly produced during the wet preservation of maize kernels due to the presence of propionic acid and a resulting acidic pH. Blending the treated maize into the compound feed, the feed pH should have shifted to a neutral, if not alkaline, pH (buffering capacity of main ingredients, incl. premix), thus enabling DONS2 formation in the experimental feed. However, in plasma of our experimental pigs, DONS2, but not DONS3, were determined. This discrepancy might be explained by the chemical instability of DONS3: with increasing pH value, starting from pH 5, this compound can be reconverted into the parent toxin DON and also into DONS2, but to a lesser extent (Schwartz et al. 2013). Therefore, the previously formed DONS3 was probably converted due to pH shifts after feed ingestion, on the one hand along the digestive tract of the pig (pH ~ 6.8 in the small intestine) and on the other hand after absorption into the blood stream (pH ~ 7.4). The latter was already reported in a study on the bioavailability of DONS in growing pigs (Paulick et al. 2015b) where DONS were administered intravenously, whereby DONS3 was particularly unstable and readily converted mainly to DON, owing to the prevailing blood pH and temperature. This is the current explanation for the difference in SoS-induced DON reduction in experimental diets and plasma of pigs.

Another metabolite of DON, DOM-1, was determined in plasma of pigs fed the *Fusarium*-toxin contaminated diet. Microbes in the digestive tract of pigs are capable of de-epoxidizing DON through the formation of DOM-1 (Goyarts and Dänicke 2006).

In the present study, the DON concentration in group DON− exceeded the guidance value of 0.9 mg DON/kg feed (Comission of European Community 2006) approximately fivefold. This DON concentration was chosen to provoke a detectable adverse effect of DON on performance of pigs in group DON- serving as a prerequisite for demonstrating the (positive) effects of the SoS-treatment. At the tested level of 4.81 mg DON/kg diet, we observed a 23% decrease in FI in the grower period compared to the CON-group. This decrease was not observed when SoS-treated DON-contaminated maize (5 g SoS/kg maize) was included in the diet. Without this DON-related drop in FI, we would not have been able to show the protective effect of the SoS treatment. An earlier study (Dänicke et al. 2008) failed to prove the efficacy of SBS treatment of DON-contaminated triticale on performance traits due to a too low DON level of the diet fed to the negative control group, although DON-concentration of feed was also markedly reduced. In the current investigation, the decrease of the performance in fattening pigs was not observed in DON groups with SoS-treatment. Here, the pigs showed similar performances compared to pigs fed the control diets. This result is in accordance with an earlier study (Paulick et al. 2018) where SoS treatment of the *Fusarium*-toxin contaminated grain restored the FI of piglets fed the DON-contaminated maize over a short period of 6 weeks.

Moreover, the aim of our study was to observe parameters of clinical biochemistry to gather information about possible influences of long-term exposure with DON or SoS on the health of fattening pigs. All parameters of the clinical biochemistry where significantly affected by the main factor time, because of growth of pigs during 10 weeks of the experiment. In a study of Kubena et al. (1989) serum cholesterol concentration was decreased in broilers fed with DON- and T-2- contaminated wheat. These findings are comparable with the results of the present study, where DON significantly decreased cholesterol concentration in serum of pigs. Probably, this effect can be explained by involvement of the liver and decreased cholesterol biosynthesis in pigs fed the DON-contaminated maize (Kubena et al. 1989). Conversely, Ghareeb et al. (2016) examined in a study increased cholesterol concentrations in broilers fed a diet contaminated with DON. The authors also suspected liver involvement as a possible reason for this increased cholesterol concentration. However, cholesterol concentrations in DON groups in the present study were only slightly different from concentrations of pigs in the CON groups and still within their physiological reference range (Kraft and Dürr 2014). Furthermore, concentrations of all other clinical biochemistry parameters, with the exception of bilirubin, were within the reference range (Kraft and Dürr 2014). Bilirubin, an indicator for liver function, was elevated (overall mean 7.80 μmol/L) compared to the reference value (up to 4.3 μmol/L) of Kraft and Dürr (2014). It has to be considered that differences in concentrations of clinical parameters might arise from differences in the age and the sex of the animals (Kixmöller 2004). In the present study, only urea concentration was significantly increased by the treatment with SoS. Moreover, data of performance of pigs demonstrated a 6.5% higher FI in groups fed the SoS-treated diets. Therefore, it is possible, that the higher urea concentration can be associated with higher protein intake due to an increased FI of pigs fed the diets treated with SoS. Tran et al. (2018b) could not detect an effect of SoS treatment on clinical chemistry when DON-contaminated maize treated with 5 mg SoS/kg maize was fed to piglets for 42 days. For urea and ALP a significant effect of vaccination was determined. However, urea and ALP concentrations were still within their physiological reference range (Kraft and Dürr 2014), and therefore, the physiological relevance of this effect is probably negligible.

To determine the health status of the pigs, further parameters were investigated. Thiamine concentration was significantly affected by time because of higher levels at the beginning of the experiment. Probably, this is explainable by change of feed when the experiment started. A short- and a long-term feeding experiment was conducted by Til et al. (1972) to investigate the effect of sulphite on the health of pigs. No adverse effects on health or mortality were determined. However, the thiamine levels in urine and liver were decreased at increased sulphite levels. The results of our study cannot confirm these findings, because here, thiamine was enhanced by SoS treatment in pigs fed the CON diets. In addition, sulphate concentration in plasma was analysed to partially investigate the sulphur metabolism in pigs after feeding with SoS-treated feedstuff. After oral ingestion, sulphite in pigs is oxidized to sulphate by the sulphite oxidase (Wyse et al. 2018). In the present study, no significant effects of DON or SoS on sulphate concentration in plasma could be detected. Further research is needed to investigate the effect of SoS treatment in diets of pigs regarding the sulphite metabolism in the body.

In conclusion, the wet preservation of *Fusarium*-contaminated maize treated with graded levels of SoS showed a marked reduction of DON in the diets over all fattening phases which was also reflected in the pigs’ plasma. Furthermore, performance of pigs could be improved by decontamination of DON by SoS treatment to a level comparable to that of the control group. In the present experiment, it could be shown that, in spite of some significant effects, long-time exposure with SoS-treated maize did not impair the health status of the fattening pigs.
